# Diversification of petal monoterpene profiles during floral development and senescence in wild roses: relationships among geraniol content, petal colour, and floral lifespan

**DOI:** 10.1007/s00442-020-04710-z

**Published:** 2020-07-25

**Authors:** K. G. Srikanta Dani, Silvia Fineschi, Marco Michelozzi, Alice Trivellini, Susanna Pollastri, Francesco Loreto

**Affiliations:** 1 Institute for Sustainable Plant Protection, National Research Council of Italy, Via Madonna del Piano 10, Sesto Fiorentino, 50019 Florence, Italy; 2 Institute of Heritage Science, National Research Council of Italy, Via Madonna del Piano 10, Sesto Fiorentino, 50019 Florence, Italy; 3 Laboratory for the Analysis and Research in Environmental Chemistry, Institute of Biosciences and Bioresources, National Research Council of Italy, Via Madonna del Piano 10, Sesto Fiorentino, 50019 Florence, Italy; 4 Institute of Life Sciences, Scuola Superiore Sant’Anna, 56124 Pisa, Italy; 5 Department of Biology, Agriculture and Food Sciences, National Research Council of Italy, Piazzale Aldo Moro 7, 00185 Rome, Italy

**Keywords:** Geraniol, Floral diversity, Floral evolution, Floral lifespan, Flower size, Floral volatiles, Isoprenoid hormones, Monoterpenes, Petal colour, Rose petals, Wild roses

## Abstract

Wild roses store and emit a large array of fragrant monoterpenes from their petals. Maximisation of fragrance coincides with floral maturation in many angiosperms, which enhances pollination efficiency, reduces floral predation, and improves plant fitness. We hypothesized that petal monoterpenes serve additional lifelong functions such as limiting metabolic damage from reactive oxygen species (ROS), and altering isoprenoid hormonal abundance to increase floral lifespan. Petal monoterpenes were quantified at three floral life-stages (unopened bud, open mature, and senescent) in 57 rose species and 16 subspecies originating from Asia, America, and Europe, and relationships among monoterpene richness, petal colour, ROS, hormones, and floral lifespan were analysed within a phylogenetic context. Three distinct types of petal monoterpene profiles, revealing significant developmental and functional differences, were identified: Type A, species where monoterpene abundance peaked in open mature flowers depleting thereafter; Type B, where monoterpenes peaked in senescing flowers increasing from bud stage, and a rare Type C (8 species) where monoterpenes depleted from bud stage to senescence. Cyclic monoterpenes peaked during early floral development, whereas acyclic monoterpenes (dominated by geraniol and its derivatives, often 100-fold more abundant than other monoterpenes) peaked during floral maturation in Type A and B roses. Early-diverging roses were geraniol-poor (often Type C) and white-petalled. Lifetime changes in hydrogen peroxide (H_2_O_2_) revealed a significant negative regression with the levels of petal geraniol at all floral life-stages. Geraniol-poor Type C roses also showed higher cytokinins (in buds) and abscisic acid (in mature petals), and significantly shorter floral lifespan compared with geraniol-rich Type A and B roses. We conclude that geraniol enrichment, intensification of petal colour, and lower potential for H_2_O_2_-related oxidative damage characterise and likely contribute to longer floral lifespan in monoterpene-rich wild roses.

**Electronic supplementary material:**

The online version of this article (10.1007/s00442-020-04710-z) contains supplementary material, which is available to authorized users.

## Introduction

Floral fragrance in roses is largely made of volatile monoterpenes, isoprenoid alcohols, and their oxidised and glycosylated (non-volatile) derivatives. Monoterpenes are stored and emitted mostly from rose petal epidermal follicles (Guterman et al. 2002). All plants make monoterpenes from geranyl diphosphate (GDP) via the plastid-localised methylerythritol phosphate (MEP) pathway (Bohlmann et al. 1998; Degenhardt et al. 2009). In addition to the MEP pathway, rose petals also possess a unique cytosolic NUDIX hydrolase pathway that almost exclusively makes geraniol, a prominent acyclic monoterpene in hybrid roses (Magnard et al. 2015; Sun et al. 2016). Peaking of floral fragrance in mature flowers enhances pollination efficiency (Dudareva and Pichersky^2006^; Parachnowitsch et al. 2012). Pollinated senescing flowers are fragrance-poor, which helps (i) divert pollinators to unpollinated (fragrance-rich) flowers, (ii) prevent floral damage due to excessive visits by pollinators and nectar robbers, and (iii) reduce seed predation (Schiestl et al. 1997; Muhlemann et al. 2006; Morris et al. 2010). Hybrid roses indeed show the highest monoterpene abundance in petals at floral maturity (Bergougnoux et al. 2007; Rusanov et al. 2011). However, petal monoterpenes are also under selection to deter floral herbivores (e.g., Schiestl 2010, Junker and Blüthgen 2010), which means that temporal changes in monoterpene abundance may not strictly reflect pollination needs of a species. Moreover, it is unclear whether petal monoterpenes by themselves perform constitutive functions to influence floral health and lifespan. Monoterpenes in leaves not only prevent herbivory but also mitigate sub-cellular oxidative damage caused by reactive oxygen species (ROS, e.g., Loreto et al. 2004). The capacity to store and emit monoterpenes is associated with longer leaf lifespan (Dani et al. 2014), and potentially also with longer floral lifespan (Rogers 2012; Dani et al. 2016).

Polyploid genotypes (bred from 8 to 10 wild species; Roberts et al. 2009; MacPhail and Kevan 2009) and multi-whorled floral phenotypes of hybrid roses likely complicate any relationship between fragrance and floral lifespan (e.g., past experiments in ornamental cut-roses; Borda et al. 2011; In et al. 2017). Instead, we explored and analysed qualitative and quantitative monoterpene profiles during floral development and senescence in 73 wild roses (57 recognized as distinct species), which are mostly diploid and single-whorled, and were grown in the same rose garden (i.e., under the same environmental conditions). Developmental changes in petal cytokinins (CKs) and abscisic acid (ABA) were quantified on the premises that large CK pools lengthen floral lifespan (Mayak and Halevy 1970; van Doorn et al. 2013), while high endogenous ABA induces senescence and curtails floral lifespan (Panavas et al. 1998; Lü et al. 2014). Hydrogen peroxide (H_2_O_2_), a non-radical reservoir of free radical ROS, was quantified with the expectation that H_2_O_2_ accumulation in plant tissues indicated senescence induction, while H_2_O_2_ depletion suggested delaying of senescence (Brennan and Frenkel 1977; Bieker et al. 2012). To integrate floral phenotypes and chemotypes for a realistic evolutionary assessment (e.g. Raguso 2008), we analysed floral size, petal colour, and floral lifespan, and assessed monoterpene profiles within the context of species-level phylogeny of wild roses. We tested the hypothesis that monoterpene enrichment lengthens floral lifespan by enhancing cytokinin abundance and by decreasing ABA and ROS levels in petals. We present a comprehensive account of how developmental changes in petal monoterpenes have diversified in wild roses. The differences in monoterpene and hormonal profiles of early- and late-senescing rose flowers (species), evolutionary links between structural and volatile floral phenotypes in wild rose lineages, and a hypothetical framework for distinct biochemical origins of specific structural classes of monoterpenes in rose petals are discussed.

## Materials and methods

### Plant material

Petals from 73 wild roses (57 species and 16 subspecies; Supplementary Table S1) were harvested from plants maintained at the Gianfranco & Carla Fineschi Rose Garden in Cavriglia, Italy (43° 31′ N, 11° 29′ E). These wild roses bloomed from April to June and were sourced from three geographic regions of origin, viz., Asia (*N *=44), Europe (EU, *N *=19), and America (*N *= 8), and two with unknown origin. Sampled roses were diploid (*N *=27), tetraploid (*N *=14), and penta- and hexaploid (*N *= 3 each), and the remaining were of unknown ploidy. Most roses were single-whorled (five-petalled, *N *= 68) and some were bi-whorled (ten-petalled, *N *= 5). Each rose species was represented by one healthy and fertile individual plant in the rose garden, implying that all species were autogamous or geitonogamous to different degrees. Each individual species acted as one biological replicate for comparative trait analyses (classified *post factum*). The empirical limitation of using just one individual per species meant that any of our results could not account for the potential diversity and plasticity in monoterpene profiles within species. However, by hosting a global collection of roses in a common garden, we were able to compare profiles among biological species under the same environmental conditions. Petal samples were harvested on mostly sunny days from 5 to 10 pseudoreplicate flowers per species (= per individual) at three time-points in the floral life cycle: (1) unopened buds with visible petals; (2) fully open mature flowers; and (3) senescent, wilting petals, prior to abscission. New flowers were sampled at each stage. Collected petals were frozen in liquid nitrogen and stored at − 80 °C until further analyses.

### Monoterpene extraction and quantification

Monoterpenes were extracted by grinding 500 mg of frozen petals (pooled from > 3 flowers per stage) in 1 mL of heptane in and immediately transferred to an air-tight head-space sampler vial and incubated at 40 °C in a sonicator, stirred overnight. The extract was centrifuged at 4000 rpm for 5 min. 200 μL of the supernatant was collected. Volatiles were identified and quantified using gas chromatography–mass spectrometry (Agilent 5977E GC–MS, Agilent Technologies, USA). A small fraction of the extract was injected in splitless mode to the GC inlet at 260 °C fitted with an HP-INNOWax column (50 m × 200 μm × 0.4 μm). Ultrapure Helium (Rivoira Gas, Italy) was supplied at the pressure of 33.6 Psi, at a total flow rate of 15 mL min^−1^ (purge flow at 50 mL min^−1^), and the column flow was 1.2 mL min^−1^. Volatile analytes were separated heating the column with the following sequence: 40 °C (1 min), increasing at 5 °C min^−1^ to 200 °C (6 min), and then to 260 °C at 10 °C min^−1^. Monoterpene standard mixtures were also prepared using high-purity components (Sigma-Aldrich). Standards were run separately and used for comparing ionisation patterns, retention times, and for preparing standard curves. Seventeen monoterpene species found in rose petals were quantified on petal fresh weight basis and classified into the following groups based on their chemical structure: (i) cyclic monoterpenes (limonene, cineole, p-cymene, and α-phellandrene, also including two bicyclic monoterpenes: α-pinene and β-pinene), (ii) acyclic monoterpene (geraniol and geraniol-derived alcohols and acetates, namely nerol, geranyl acetate, and β-citronellol). These are also termed “geraniols” hereafter, and species were also classified on the basis of geraniols (G) content: high (G1000, i.e., storing more than 1000 nmol geraniols gFW^−1^); medium (G100, i.e., storing more than 100–1000 nmol geraniols gFW^−1^); or low (G10, i.e., emitting 10–100 nmol geraniols gFW^−1^); and (iii) other acyclic monoterpenes (linalool, myrcene, and β-ocimene). Non-volatile monoterpene conjugates were not quantified.

### Floral phenotyping and phylogenetic comparison

Species were categorised based on petal colour–pale pink (mixed white), pink (dark pink and purple), white, and yellow. Monoterpene profile types, geraniol abundance, and petal colour were linked to the phylogeny of 39 rose species, whose relatedness were discerned from published phylogenies of wild roses (Bruneau et al. 2007; Fougère-Danezan et al. 2015). Flower size and floral lifespan were estimated in all of these roses except *R.sicula, R.nutkana, R.multibracteata,* and *R.acicularis*. The sample size was 6–10 flowers per species for floral size, and 6–25 flowers per species for floral lifespan. Size was represented by floral circumference measured in situ as well as calculated from photographs (using ImageJ software v1.51 w). Floral lifespan, from unopened bud stage to petal abscission, was monitored during two flowering seasons. Individual flower buds per species were tagged at early unopened bud stage and floral lifespan was calculated as the time (in days) required for individual flowers to senesce and die (shed petals).

### Extractable hydrogen peroxide (H_2_O_2_) quantification by peroxidase assay

Sixty mg of petals were ground in liquid nitrogen with 5% polyvinyl pyrrolidone (PVP), and the ground tissue was suspended in 2 mL of 1 N perchloric acid. The extract was cold centrifuged for 15 min. Five hundred μL of the supernatant were aliquoted and an equal volume of acidic phosphate buffer (pH 5) was added. Fifty μL were treated with ascorbate oxidase for 15 min at room temperature to remove ascorbates (and neutralise the extract). A reaction mixture comprising 3-dimethylamino benzoic acid (DMAB), and 3-methyl-2-bonzothiazolinone hydrazine (MTAB), and phosphate buffer was placed in plate-microcuvettes. The neutralised extract and horse-radish peroxidase were added to the reaction mixture. Absorbance was monitored continuously at 590 nm, where an absorption peak is known for the deeply purple coloured product of the oxidative coupling between MTAB and DMAB in the presence of H_2_O_2_ and peroxidase (Infinite 200 PRO Plate Reader, Tecan Instruments, CH). Petal H_2_O_2_ was quantified by generating a standard curve with known amounts of pure H_2_O_2_. The quantification was done in a subset of 14 rose species representing Types A, B, and C monoterpene profiles based on tissue availability (see results). Each profile type included different petal colours and geraniol abundances (G10 to G1000). The 14 species were *Rosa foliolosa, R. gallica,* and *R. nutkana* (all pink, all G1000), *R. xanthina* (yellow, G1000)*, R. arvensis* (white, G100), and *R. bracteata,*(white, G10) all Type A (*N *= 6); *R. longicuspis* (white, G1000)*, R. chinensis mutabilis and R. canina* (pink, G1000), and *R. laevigata* (white, G10), all Type B, (*N *= 4); *R. villosa* (pink, G100)*, R. foetida* (yellow, G100)*, R. banksiae,* and *R. soulieana* (white, G10), all Type C (*N *= 4).

### Abscisic acid and cytokinin quantification

Abscisic acid (ABA) was extracted from 100 mg fresh weight of petals per sample (tissues previously frozen in liquid nitrogen and stored at − 80 °C) in distilled water overnight at 4 °C (water: tissue ratio = 10:1 v/w). ABA was determined by an indirect enzyme-linked immunosorbent assay (ELISA) using DBPA1 monoclonal antibody, raised against S (+)-ABA (Vernieri et al. 1989; Trivellini et al. 2015). ABA was quantified in all rose species used for monoterpene profiling.

Cytokinins (CKs) were extracted in acidified aqueous methanol, purified by two solid-phase extraction (SPE) steps and subsequently measured with LC–MS/MS (after Schäfer et al. 2014). Trans-zeatin (tZ), trans-zeatin riboside (tZR), trans-zeatin riboside-O-glucoside (tZROG), and cis-zeatin riboside-O-glucoside (cZROG) were chromatographically separated on a Zorbax Eclipse XDB-C18 column (50 × 4.6 mm, 1.8 µm) fitted to an Agilent 1200 HPLC system (Agilent Technologies, USA). LC was coupled to an API 6500 tandem mass spectrometer (AB Sciex, Darmstadt, Germany) and deuterated standards of each compound were used as reference for quantification. The CK assay was done in a subset of 9 representative species, where three species each represented Types A, B, and C monoterpene profiles, included both white and coloured petals, and both geraniol-poor and geraniol-rich species. The 9 species were *Rosa gallica* (pink, G1000)*, R. nutkana,*(pink, G1000) and *R. bracteata* (white, G10), all Type A; *R. longicuspis* (white, G1000) *R. chinensis mutabilis* (pink, G1000), and *R. laevigata* (white, G10), all Type B; *R. villosa* (pink G100)*, R. foetida* (yellow, G100), and *R. banksiae* (white, G10), all Type C.

### Statistical analysis

The significance of normal distribution of total monoterpene content in petals was tested (inclusive of all roses, all three life-stages) using the Shapiro–Wilk (SW) test and they were found to be normally distributed (*p *< 0.01). Outliers were identified and excluded from further analysis using Grubb’s test. Equality of variances among life-stages and among geographic groups was tested using Levene’s test (*α* = 0.05). Significance of differences among means was tested using a General Linear Model (multifactor nested ANOVA for repeated-measures design). Geographic region (2 degrees of freedom, dof), monoterpene profile type (2 dof), life-stage (2 dof), and ploidy (4 dof) were the main factors. Differences in means among types, regions, and stages were analysed using Tukey’s test (*α* = 0.05) with conservative letter coding whenever possible. If variances were unequal, the Games–Howell test was applied. Significance of regressions (linear) among monoterpene abundance, extractable H_2_O_2_, and phytohormone content were tested within and among 3 geographic groups (America, Asia, and EU), 3 life-stages (unopened buds, fully open mature flowers, and senescing flowers), and 3 monoterpene profile types (A, B, and C). The consistency (trueness) of match between monoterpene profiles and their geographic group was tested by Discriminant Analysis, a technique where two or more groups, clusters, populations are known a priori, to which one or more new observations are matched. All statistical tests were conducted using Minitab statistics software v18 (Minitab Inc, USA).

## Results

### Petal monoterpene profiles over flower lifespans

**Fig. 1:**
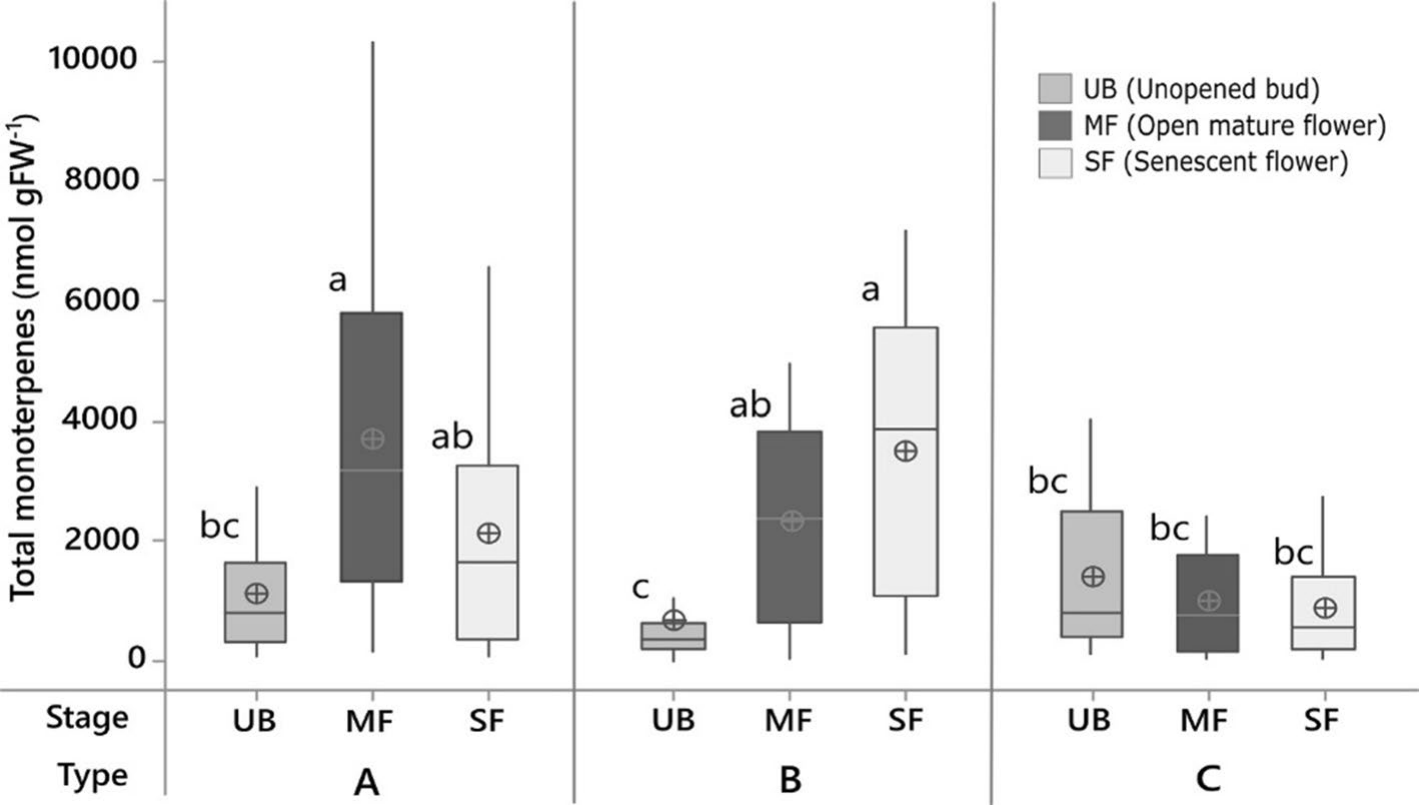
Petal monoterpene profiles during floral development and senescence in wild roses. Three distinct profile types (Types A, B, and C) are shown. Monoterpene concentration is quantified in petals sampled at three different floral life-stages—unopened bud (UB); open mature flower (MF); and senescent flower (SF). The box for each life-stage includes the mean (a circle with a plus mark), the median (the middle horizontal line), and the box spans lower and upper quartiles. The whiskers span the full data range. Equality of means is tested using a general linear model repeated-measures ANOVA followed by Tukey’s test (*α* = 0.05, *N *= 71, 30 Type A, 27 Type B, 8 Type C). Significant differences do not share letter coding

**Fig. 2:**
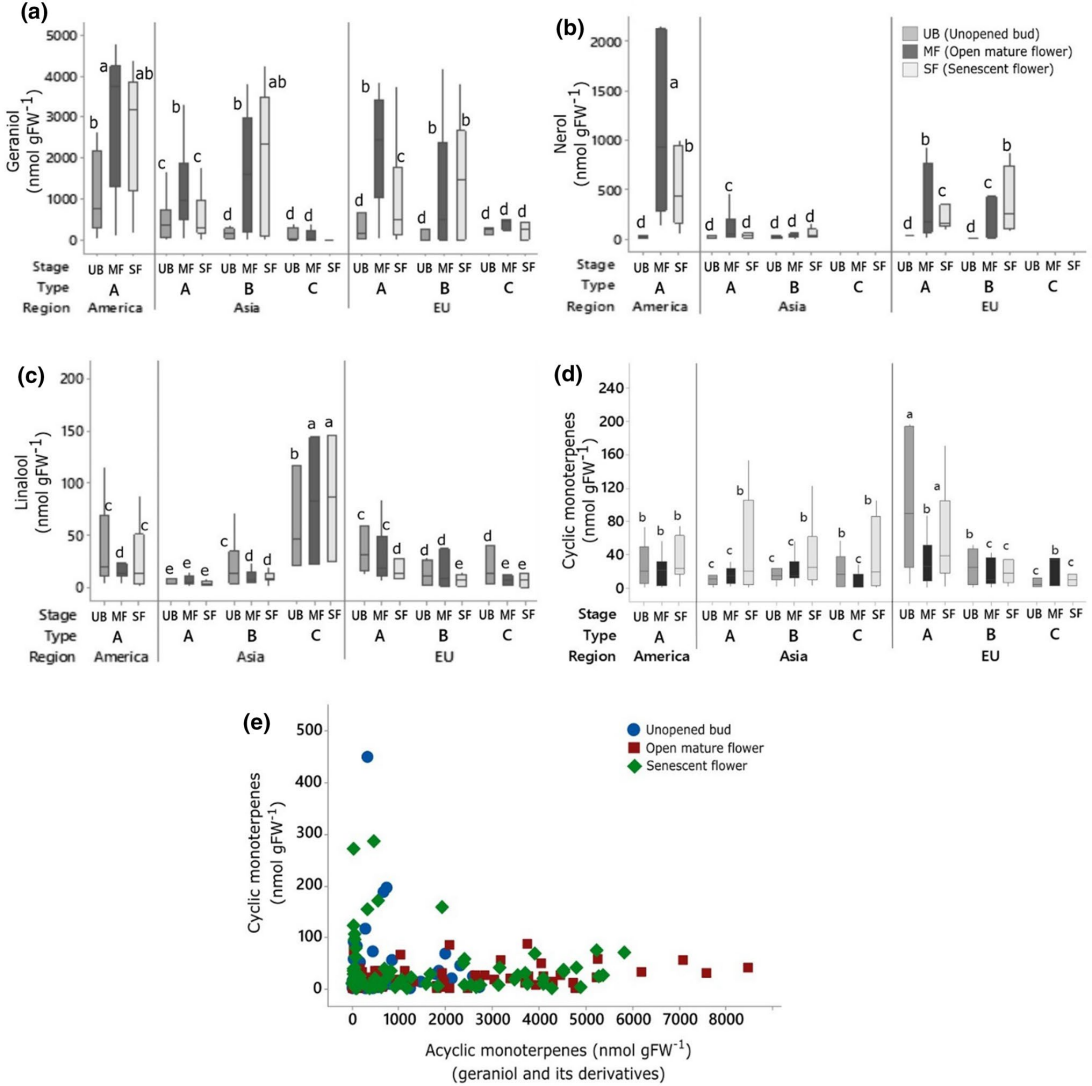
Quantitative changes in major monoterpenes in wild rose petals at three floral life-stages: (**a**) Geraniol, (**b**) Nerol, (**c)** Linalool, (**d**) Cyclic monoterpenes. Roses belong to three geographic regions and represent three profile types (as in Fig. 1). Data box for each life-stage includes the mean, the median with lower and upper quartiles, and the whiskers cover the full range. Equality of means is tested using GLM ANOVA followed by Games–Howell test, without assuming equal variance (*α* = 0.05, *N* as in Fig. 1). Significant differences do not share letter coding. (**e**) Scatter plot showing tendency towards mutual exclusivity between cyclic and acyclic monoterpenes at all floral life-stages

Developmental changes in petal monoterpene profiles in wild roses showed three significantly different profiles hereafter called *Types* (Fig. 1). Type A showed significantly higher total monoterpene abundance at floral maturity than in unopened buds. Type B showed a significant increase in monoterpenes from unopened buds to floral maturity and further accumulation of monoterpenes during senescence. Type C showed a decline in monoterpenes from bud stage to senescence although difference among stages was not significant (Fig. 1). Geraniol was the most prominent monoterpene in all roses, often ≥ 2000 nmol gFW^−1^ in geraniol-rich Type A and B roses (> 80% of the total monoterpenes) (Fig. 2a). Type C roses were significantly poorer of total monoterpenes (< 1000 nmol gFW^−1^ at any stage) and specifically poorer of geraniol compared with Type A and B (Fig. 2a). Both geraniol and nerol significantly increased in mature flowers and then decreased in senescent flowers of all Type A roses (Fig. 2a, b). The decrease was significant in Type A roses except in American roses where such a decrease was evident but not significant. Levels of geraniol stayed high in senescent petals of all Type B roses (Fig. 2a). Levels of nerol were highest in American roses (Fig. 2b). EU Type B roses significantly accumulated nerol even during senescence, while no significant variation in nerol abundance was detected among floral life-stages of Asian roses. Type C roses showed no detectable content of nerol (Fig. 2b). Linalool (the main component of acyclic monoterpenes not derived from geraniol) abundance was highest in Type C Asian rose (~ 150 nmol gFW^−1^; Fig. 2c). Cyclic monoterpenes (mainly α-pinene, β-pinene, limonene, cineol, and p-cymene) were low (comparable to linalool) and did not change over the floral lifespan in American Type A roses, and tended to decrease along floral lifespan in EU Type A and B roses, whereas all Asian roses showed a significant increase in cyclic monoterpenes in senescing petals (Fig. 2d). No unique monoterpene was associated with any species (note: limited genetic representation in the rose garden). A scatter plot of absolute abundance of acyclic and cyclic monoterpenes (stage-wise) showed an L-shaped distribution, suggesting a trade-off between these two classes of monoterpenes (Fig. 2e). However, acyclic-to-cyclic monoterpene absolute ratios were 100:1 in 83% of all observations (typically in mature petals of Type A and B roses), 1:1 in 13% of observations (either in buds or senescent petals of all Types), and 1:10 in mere 4% of all observations (typically Type C roses).

### Petal monoterpenes and petal colour association in wild roses

**Fig. 3:**
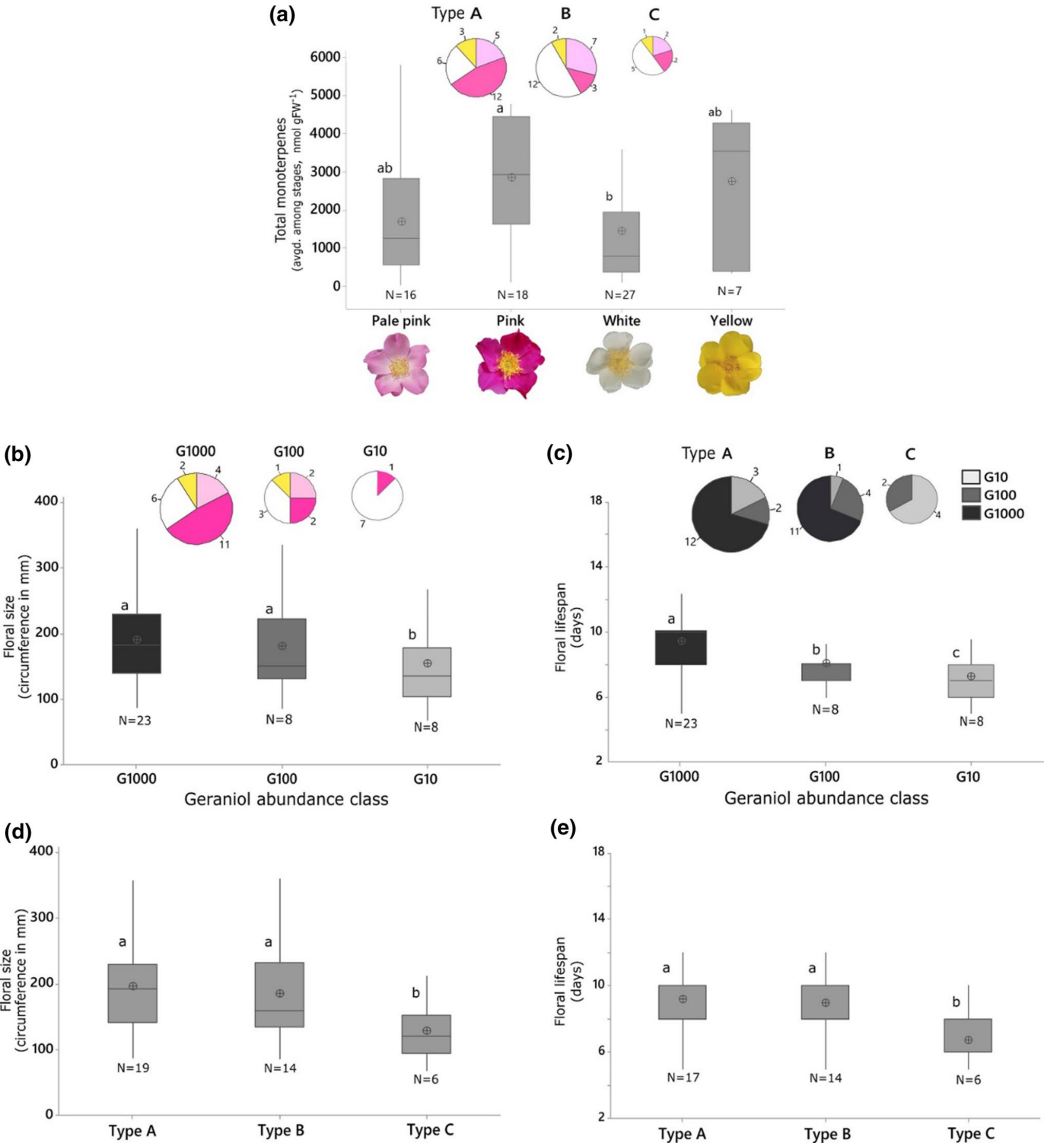
Petal monoterpene profiles and floral phenotype in wild roses. **a** Monoterpene content as a function of petal colour, (**b**, **d**) Floral size and (**c**, **e**) Floral lifespan in relation to geraniol abundance and monoterpene profile types, respectively. The size of pie charts in (**a–c**) is proportional to the sample size. Each data box includes the mean, the median with lower and upper quartiles, and the whiskers cover the full data range. Equality of means is tested using GLM ANOVA followed by Tukey’s test (at *α* = 0.05, *N *≥ 39 species, except 3 (**e**). Significant differences do not share letter coding

Species with white petals contained the least levels of monoterpenes, while yellow and pale-pink petals contained intermediate levels of monoterpenes and pink petals contained the highest levels of monoterpenes (Fig. 3a). Flowers of geraniol-poor species (G10) were significantly smaller (Fig. 3b) and shorter lived (Fig. 3c) compared with G100 and G1000 species. When analysing floral size and floral lifespan by rose Type (A, B, C), we found that petals of geraniol-rich Type A and B roses were bigger and lasted longer compared with small, monoterpene-poor Type C roses (Fig. 3d, e).

**Fig. 4:**
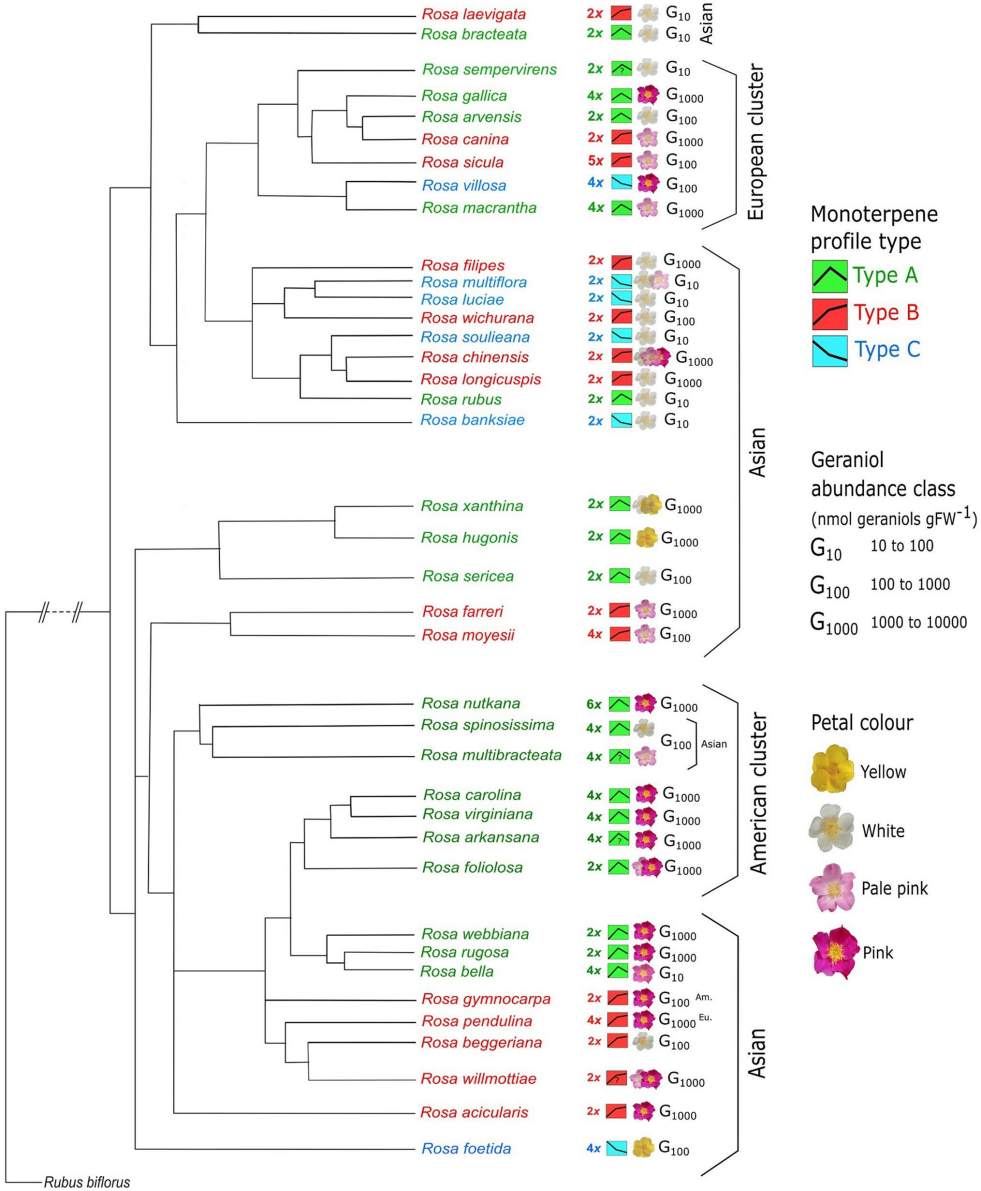
Phylogenetic associations among monoterpene profile types, petal colour and geraniol abundance in wild roses. Monoterpene profile types (A, B, and C), petal colour (white, pale pink, pink, and yellow), and geraniol concentration (G10, G100, and G1000) are overlaid on a phylogenetic tree of wild roses. The phylogeny is assembled in a simplified form from Bruneau et al. (2007) and Fougère-Danezan et al. (2015)

In nearly 60% of the species, developmental petal monoterpene profiles showed a true correspondence with geographic grouping of roses in the discriminant analysis. The proportion of true grouping was high in American (0.8, all Type A as already mentioned) and Asian roses (0.64, mostly Type B), compared with European roses (0.5). Assembled phylogenies placed American and European roses in separate monophyletic clusters and Asian roses formed different groups (Fig. 4). Densely pink or purple, Asian Type A roses (e.g., *R. webbiana, R. rugosa*) were closely related to pink, geraniol-rich (G1000) American Type A roses. All American roses were pink petalled and geraniol-rich. Type C condition was rare, typically associated with white-petalled and geraniol-poor (G10) roses (Fig. 2a). Type C condition was seen in *R. banksiae* (Section Banksianae, white) and *R. foetida* (Section Pimpinellifolia, yellow), which are among early-diverging roses (see Fougère-Danezan et al. 2015). Some Type C roses were also part of a single Asian cluster (Section Synstylae) which was otherwise geraniol-rich and Type B (*R. chinensis, R. filipes,* and *R. longicuspis*). Early-diverging roses also included *R. bracteata* (Section Bracteatae, white, Asian, Type A), and *R. laevigata* (Section Laevigatae, white, Asian, Type B), which were all geraniol-poor (G10, Fig. 4).

### Petal H_2_O_2_ and phytohormones

**Fig. 5:**
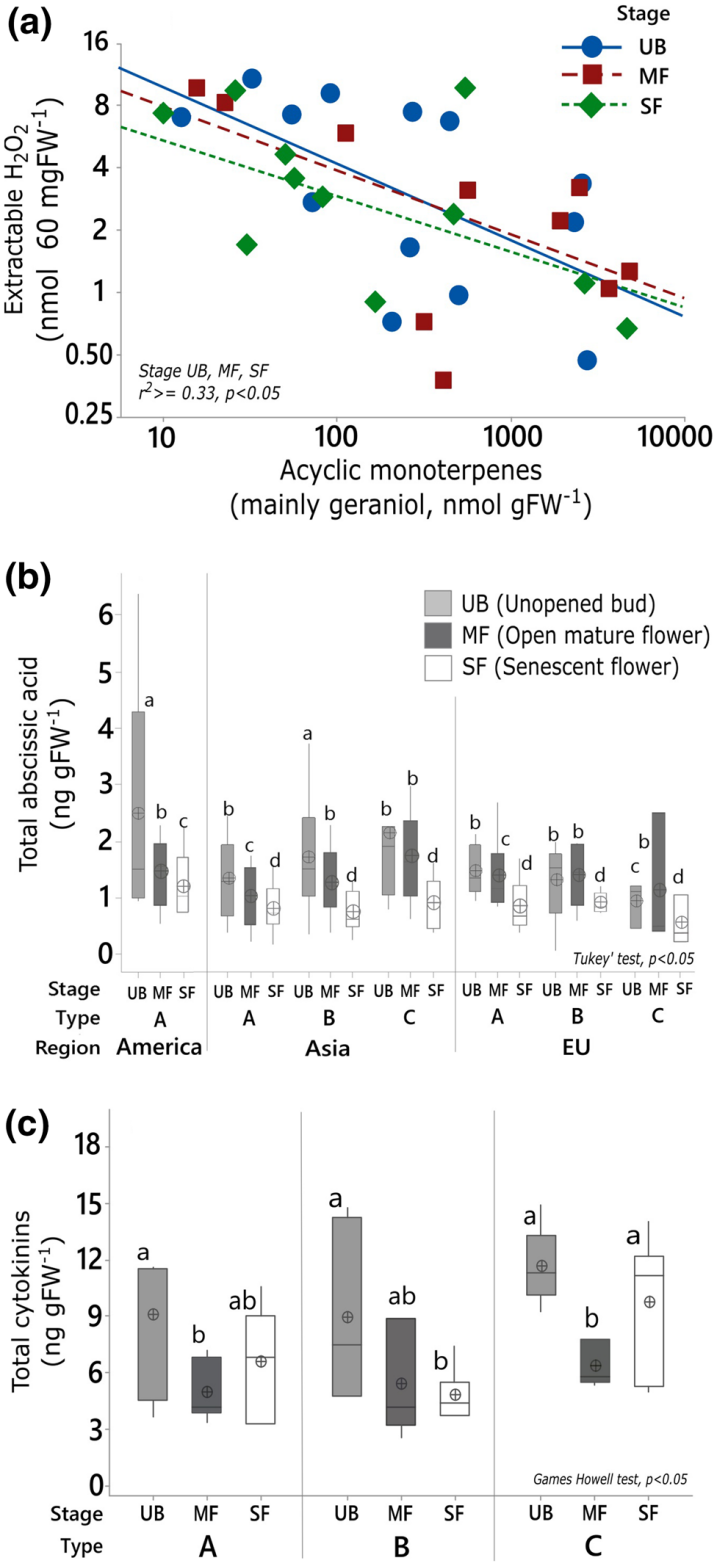
**a** Regression between the concentrations of extractable hydrogen peroxide (H_2_O_2_) and acyclic monoterpenes (geraniols), abundance of **b** Total abscisic acid (ABA) and **c** Total cytokinins (CKs) in wild rose petals. Regression fits in **a** for UB, MF, and SF are all significant (*α* = 0.05). Equality of means in (**b**, **c**) is tested using GLM ANOVA followed by Tukey’s test for ABA (*α* = 0.05), and Games–Howell test for CKs (*α* = 0.05). Each data box includes the mean, the median with lower and upper quartiles, and the whiskers cover the full data range. Significant differences do not share letter coding. ABA is quantified in all species. Names of species used for H_2_O_2_ (*N *= 14) and CKs (*N *= 9) analyses are given under methods

The regression between acyclic monoterpene content (mainly geraniol) and extractable H_2_O_2_ showed a significant negative slope at all life-stages (Fig. 5a). Unopened buds contained significantly higher ABA than other stages in all roses, except in Type C (Fig. 5b). ABA levels decreased significantly from mature to senescent flowers in all roses (Fig. 5b). Buds had higher total CKs than other stages in all roses (Fig. 5c). Nearly 90% of total CK was made of inactive tZROG and cZROG, while the remaining 10% was made of active tZ and tZR. The active fraction of CKs decreased significantly only in mature Type C roses (Fig. S1).

## Discussion

Lifetime petal monoterpene profiles in wild roses reveal three types of species (Fig. 1), viz., those that maximise monoterpene abundance in fully open mature flowers (Type A), in senescent old flowers (Type B), and in unopened floral buds (Type C). Type A and B roses are typically rich in geraniols, while Type C roses are geraniol-poor and overall monoterpene-poor (Fig. 2a, b).

Significantly high monoterpene abundance in mature petals of Type A and B roses supports the premise that enhanced volatile fragrance in mature flowers may provide pollination benefits in such species (Dudareva and Pichersky^2006^; Parachnowitsch et al. 2012). However, monoterpene abundance can reach their highest levels also in immature (Type C) or even senescent petals (Type B), pointing to other developmental, functional, and seasonal constraints not restricted to pollination (e.g., Raguso et al. 2006; Weiss et al. 2016). Since wild roses bloom from April (cool spring) to July (hot summer), a seasonal temperature-induced selective enhancement of geraniols cannot be ruled out in some Type A and B species (as in leaves; Staudt and Bertin, 1998). Monoterpene-rich senescent petals (Type B) can deter florivores and seed predators (Pichersky and Gershenzon 2002; Junker and Blüthgen 2010). Senescent Type B petals may not actively synthesize monoterpenes, rather utilise a large vacuolar pool of non-volatile glycosylated monoterpene conjugates found in some roses, not quantified in our study (can be ~ 20% of total monoterpenes in hybrid roses; Oka et al. 1999). Since glycosylation is required when petals are rich in geraniols just before floral maturation (Ackermann et al. 1989), we expect geraniol-poor Type C roses to contain little or no glycosylated monoterpenes. In this respect, a handful of geraniol-poor (G10) Type A and B roses (e.g., *R. bracteata, R. laevigata, R. bella, R. rubus,* and *R. sempervirens*) can be viewed as monoterpene-poor Type C species. Monoterpene abundance in senescent Type A roses decreases likely because of a hormonal intervention similar to that in *Petunia* flowers, where fertilised gynoecium releases ethylene to down-regulate petal volatile synthesis (Underwood et al. 2005; Clark et al. 2009). Phylogenetic affinity of some Type B (G1000) Asian roses with the rare Type C (G10) species (Fig. 4) hints that a mechanism to down-regulate monoterpene synthesis in senescing flowers is not required in monoterpene-poor Type C roses and, perhaps has not evolved in Type B roses.

The strong positive correlation and phylogenetic association between monoterpene enrichment and petal colour intensification (Fig. 3a, 4) is consistent with genome sequencing data that show the co-regulation of pigment and fragrance biochemical pathways in rose petals (*Rosa chinensis*; Raymond et al. 2018). Pleiotropic linkages between scent and pigment are probably due to common regulatory elements and transcription factors rather than structural genes. While an association between petal pigments and floral aromatics (benzenoids and phenylpropanoids specifically) was anticipated, the fact that even monoterpenes increase in brightly coloured petals suggests a wider functional conjunction between fragrance and colour among species, and even among broader aggregates of co-inhabiting genera (Kantsa et al. 2017). Nonetheless, all monoterpene profile types (A, B, and C) and geraniol abundance classes (G10, G100, and G1000) may be observed in roses with all petal colours, highlighting divergent constraints on fragrance and petal pigments in roses.

Reduction in floral conspicuousness (size and fragrance-wise) can be a derived trait in many angiosperm families (e.g., Endress 2011). If true also for roses, then less conspicuous, small and geraniol-poor Type C flowers (Fig. 3b, d) should be evolutionarily advanced or derived. However, the Type C condition is not monophyletic and one cannot ignore that the earliest-diverging and phylogenetically isolated rose species are also geraniol-poor (often Type C) and those species are not ‘younger’ than others (Fig. 4). Therefore, Type C is most likely the ancestral condition in roses.

Pollinator behaviours in wild roses are mostly unknown and the extent of self-compatibility is highly unpredictable (MacPhail and Kevan 2009). Limited available evidence suggests that polyploid roses tend to be more self-compatible than diploid roses and such flexibility is one of the genetic consequences of polyploidy (Ueda and Akimoto 2001). Since polyploidy is derived many times and need not always lead to monoterpene enrichment during rose diversification (Fig. 4), and since strict-autogamy may shorten floral lifespan (Weber and Goodwillie 2013), we postulate that polyploid species that bear geraniol-rich flowers may show “delayed” self-compatibility, i.e., younger mature flowers are generally cross-pollinated and older unpollinated flowers may become self-compatible (e.g., Arathi et al. 2002; MacPhail and Kevan 2009). This may partly contribute to longer floral lifespans of geraniol-rich (big) Type A and B roses compared with short-lived flowers of geraniol-poor (small) Type C roses (Fig. 3c, e).

Negative regression between H_2_O_2_ and monoterpene abundance at any stage of floral development and in any type of rose (Fig. 5a) supports the premise that monoterpene-rich petals undergo less ROS and oxidative stress with gains in floral longevity. We had also expected abundances of petal monoterpenes to correlate with those of ABA (low when monoterpenes are high) and CKs (high when monoterpenes are high) due to their proposed interactive influence on leaf and petal senescence (Dani et al. 2016). Partly contrary to expectations, ABA levels are significantly high in floral buds compared with senescent petals in all roses irrespective of their monoterpene status (Fig. 5b). Thus, immature petals seem to behave as ABA sinks and senescent petals as ABA sources (as in leaves; Jeschke et al. 1997). A spike in petal ABA expected before senescence (Mayak and Halevy 1972; Page-Degivry et al. 1991) is noticeable only in the mature petals of geraniol-poor Type C roses (Fig. 5b), suggesting early onset of senescence. Type C buds contain high levels of total CKs (Fig. 5c) and trans-zeatin (Fig. S1), indicating fast floral maturation, which is consistent with their traits of early accumulation of monoterpenes in buds (Fig. 1) and short floral lifespan (Fig. 3e).

**Fig. 6:**
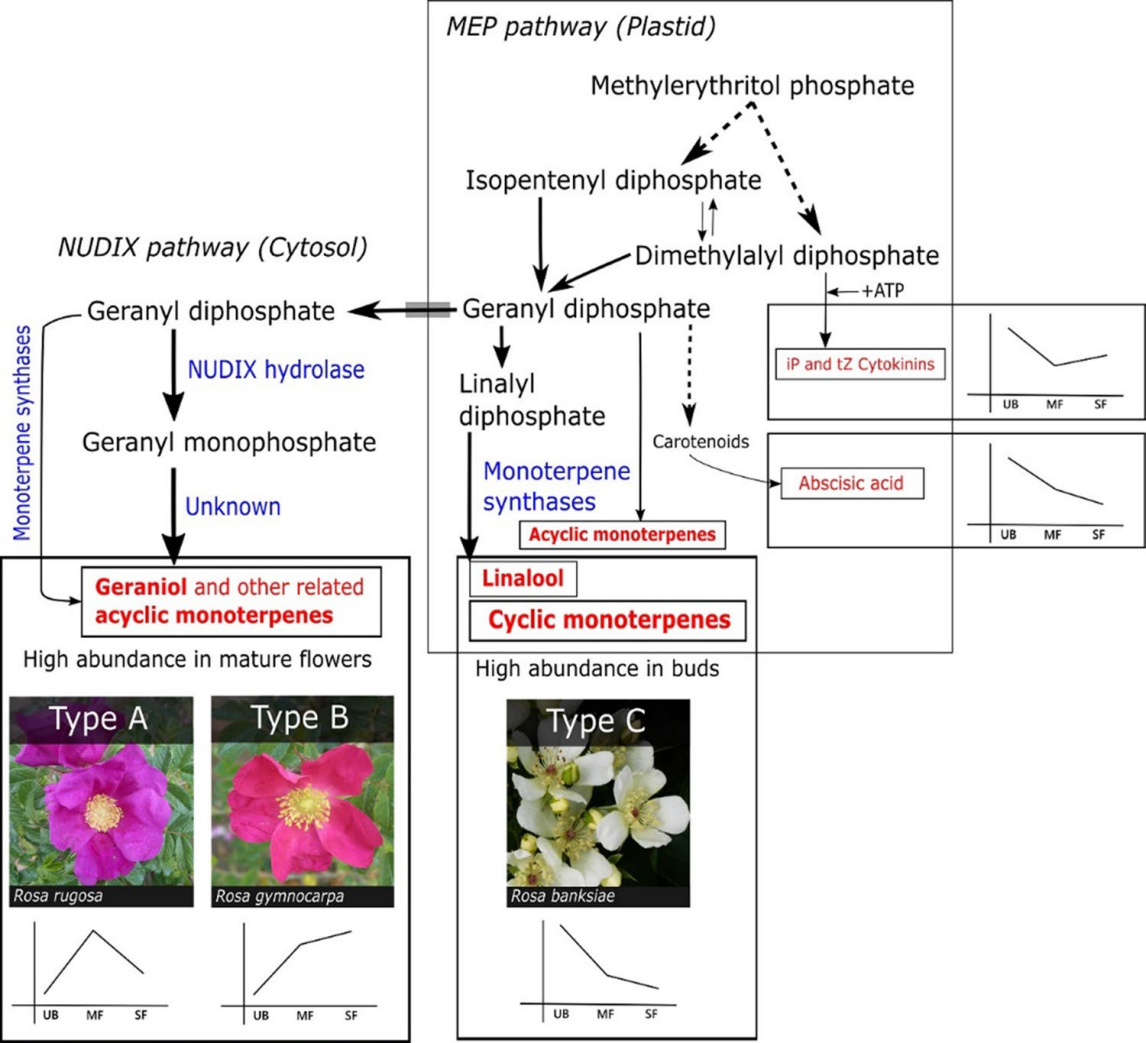
Monoterpene biosynthesis in wild rose petals. The methylerythritol phosphate (MEP) pathway in plastids and the newly elucidated NUDIX hydrolase pathway in cytosol are responsible for monoterpene synthesis in rose petals. Geraniol-poor Type C roses likely possess only the conventional MEP pathway, which makes all kinds of monoterpenes at low levels. In geraniol-rich Type A and B roses, floral maturity coincides with geraniol enrichment by cytosolic NUDIX hydrolase pathway, which is likely absent in Type C roses. Cytokinins (isoprenoid zeatins and their conjugates Fig. S1), cyclic monoterpenes, and linalool are made by the MEP pathway during early floral development in most roses

Distinct temporal trends of individual monoterpenes (Fig. 2) suggest distinct biochemical and developmental constraints on their syntheses. It is known that geranyl diphosphate (GDP) directly gives rise to geraniols and other acyclic monoterpenes, whereas conversion of GDP to linalyl diphosphate (LDP) is necessary for cyclic monoterpene synthesis (Degenhardt et al. 2009; Fig. 6). Thus, the L-scattering between geraniols and cyclic monoterpenes (Fig. 2e) suggests a choice between GDP-derived acyclic geraniols and LDP-derived cyclic monoterpenes in rose petals. Geraniol-rich hybrid rose petals are linalool-poor despite possessing a linalool synthase (Magnard et al. 2018), and geraniol-poor Type C (Asian) roses are significantly richer in linalool compared with geraniol-poor Type A and B roses (Fig. 2c). However, acyclic geraniols are often 100 times more abundant than other monoterpenes in most roses, and this dominance appears to hide a clear trade-off between acyclic and cyclic monoterpene synthesis (Fig. 2e). Rose petals likely use conventional monoterpene synthases and the MEP pathway to synthesize linalool and cyclic monoterpenes (Fig. 6), as seen in *Arabidopsis, Petunia,* and *Antirrhinum* flowers (Dudareva and Pichersky, 2006; Muhlemann et al. 2014). A geraniol-specific monoterpene synthase (potentially a cytosolic NUDIX hydrolase, Fig. 6; Magnard et al. 2015) is potentially responsible for geraniol enrichment in Type A and B roses. If monoterpene-poor Type C is the ancestral condition (as discussed), then it follows that co-opting of NUDIX hydrolase for geraniol synthesis is absent in early roses (Type C) and evolved later in Type A and B roses.

In summary, our observations in wild roses show the following:

(i) Abundance of acyclic monoterpenes (geraniols) is maximised at floral maturity in many wild roses and geraniol enrichment is associated with significantly smaller H_2_O_2_ pools, indicating lower potential for ROS-induced damage and better floral health.
(ii) Species that maximise monoterpene level in unopened buds (Type C) are rare, always geraniol-poor, small, white-petalled, and short-lived compared with common, typically geraniol-rich, pink petalled Type A and B roses. The geraniol-poor condition characterises most of the early-diverging and phylogenetically isolated species with short-lived flowers, supporting the notion that the last common ancestor of all modern species roses had monoterpene-poor, white-petalled, and short-lived flowers.
(iii) Qualitative and quantitative petal monoterpene profiles can act as metabolic markers for floral longevity in rose breeding.

## Electronic supplementary material

Below is the link to the electronic supplementary material.

Supplementary material 1 (DOCX 309 kb)
