# Supplementary material for: Diversification of petal monoterpene profiles during floral development and senescence in wild roses: relationships among geraniol content, petal colour, and floral lifespan
Source: Oecologia. 2020 Jul 25;197(4):957–69. doi: 10.1007/s00442-020-04710-z (PMC8591013; doi:10.1007/s00442-020-04710-z)
Supplement: Supplementary file 1 — Supplementary material 1 (DOCX 309 kb) [file 442_2020_4710_MOESM1_ESM.docx]

**Supplementary material**

**Diversification of petal monoterpene profiles during floral development and senescence in wild roses: relationships among geraniol content, petal colour, and floral lifespan**

**K G Srikanta Dani ^a*^, Silvia Fineschi ^b^, Marco Michelozzi ^c^, Alice Trivellini ^d^, Susanna Pollastri ^a^, Francesco Loreto ^e*^**

^a^ Institute for Sustainable Plant Protection, National Research Council of Italy, Via Madonna del Piano 10, Sesto Fiorentino 50019, Florence, ITALY

^b^ Institute of Heritage Science, National Research Council of Italy, Via Madonna del Piano 10, Sesto Fiorentino 50019, Florence, ITALY

^c^ Laboratory for the Analysis and Research in Environmental Chemistry, Institute of Biosciences and Bioresources, National Research Council of Italy, Via Madonna del Piano 10, Sesto Fiorentino 50019, Florence, ITALY

^d^ Institute of Life Sciences, Scuola Superiore Sant'Anna, Pisa 56124, ITALY

^e^ Department of Biology, Agriculture and Food Sciences, National Research Council of Italy, Piazzale Aldo Moro 7, 00185 Rome, ITALY

*Corresponding authors

| **Table S1: List of wild rose species profiled for petal monoterpenes** | | |
| --- | --- | --- |
| **Rose species** | **Geographic affiliation** | **Taxonomic section** |
| *R. spaldingii* | America | Cinnamomeae DC. |
| *R. arkansana* | America | Cinnamomeae DC. |
| *R. gymnocarpa* | America | Cinnamomeae DC. |
| *R. melina* | America | Cinnamomeae DC. |
| *R. foliolosa* | America | Cinnamomeae DC. |
| *R. nutkana* | America | Cinnamomeae DC. |
| *R. virginiana* | America | Carolinae Crép. |
| *R. carolina* | America | Carolinae Crép. |
| *R. britzensis (Koehne)* | Asia | Caninae DC. |
| *R. rubus* | Asia | Synstylae DC. |
| *R. moschata* | Asia | Synstylae DC. |
| *R. luciae* | Asia | Synstylae DC. |
| *R. wichurana* | Asia | Synstylae DC. |
| *R. sinowilsonii* | Asia | Synstylae DC. |
| *R. longicuspis* | Asia | Synstylae D.C. |
| *R. mulliganii* | Asia | Synstylae DC. |
| *R. soulieana* | Asia | Synstylae DC. |
| *R. filipes* | Asia | Synstylae DC. |
| *R. gentiliana* | Asia | Synstylae DC. |
| *R. multiflora japonica* | Asia | Synstylae DC. |
| *R. moschata nepalensis (R. brunonii)* | Asia | Synstylae DC. |
| *R. multiflora cathayensis* | Asia | Synstylae DC. |
| *R. rugosa rubra* | Asia | Cinnamomeae DC. |
| *R. moyesii rosa* | Asia | Cinnamomeae DC. |
| *R. beggeriana* | Asia | Cinnamomeae DC. |
| *R. bella* | Asia | Cinnamomeae DC. |
| *R. willmottiae* | Asia | Cinnamomeae DC. |
| *R. multibracteata* | Asia | Cinnamomeae DC. |
| *R. pratii* | Asia | Cinnamomeae DC. |
| *R. acicularis nipponensis* | Asia | Cinnamomeae DC. |
| *R. rugosa repens alba* | Asia | Cinnamomeae DC. |
| *R. rugosa* | Asia | Cinnamomeae DC. |
| *R. webbiana* | Asia | Pimpinellifoliae DC. |
| 'Helen Knight' | Asia | Pimpinellifoliae DC.  Bred by [F.P. Knight](https://www.helpmefind.com/gardening/l.php?l=7.8276) (United Kingdom, 1966) |
| *R. xanthina spontanea* | Asia | Pimpinellifoliae DC. |
| *R. xanthina hugonis (Canary bird)* | Asia | Pimpinellifoliae DC. |
| *R. sericea pteracantha* | Asia | Pimpinellifoliae DC. |
| *R. hugonis* | Asia | Pimpinellifoliae DC. |
| *R. spinosissima altaica* | Asia | Pimpinellifoliae DC. |
| *R. foetida bicolor* | Asia | Pimpinellifoliae DC. |
| *R. pimpinellifolia lutea* | Asia | Pimpinellifoliae DC. |
| *R. pimpinellifolia* | Asia | Pimpinellifoliae DC. |
| *R. farreri persetosa* | Asia | Pimpinellifoliae DC. |
| *R. foetida* | Asia | Pimpinellifoliae DC. |
| *R. chinensis mutabilis* | Asia | Chinenses DC |
| *R. bracteata* | Asia | Bracteatae |
| *R. laevigata* | Asia | Laevigatae |
| *R. banksiae normalis* | Asia | Banksianae |
| *R. pendulina pyrenaica* | EU | Cinnamomeae DC. |
| *R. sicula* | EU | Caninae DC. |
| *R. horrida* | EU | Caninae DC. |
| *R. villosa pomifera* | EU | Caninae DC. |
| *R. marginata* | EU | Caninae DC. |
| *R. canina inermis Clou* | EU | Caninae DC. |
| *R. agrestis* | EU | Caninae DC. |
| *R. canina* | EU | Caninae DC. |
| *R. canina Jacquart* | EU | Caninae DC. |
| *R. myriacantha* | EU | Pimpinellifoliae DC. |
| *R. spinosissima syn. R. pimpinellifolia* | EU | Pimpinellifoliae DC. |
| *R. sempervirens* | EU | Synstylae DC. |
| *R. sublaevis (gallica L. × arvensis)* | EU | Synstylae DC. |
| *R. arvensis* | EU | Synstylae DC. |
| *R. phoenicia* | EU | Synstylae DC. |
| *R. marcyana (gallica × tomentosa)* | EU | Gallicanae |
| *R. sancta (R. richardii)* | EU | Gallicanae |
| *R. macrantha* | EU | Gallicanae |
| *R. gallica sylvatica* | EU | Gallicanae |
| *R. gallica complicata* | EU | Bred by Unknown (before 1800) |
| 'Glory of Edzell' | Asia | Bred by Unknown Hyb. Spinosissima |
| Coryana (syn. *Rosa* x *coryana* Hurst) | Asia | Bred by Dr. C. C. Hurst (circa 1926) |
| Dunwich Rose | Asia | Bred by Unknown (1950) Hyb. Spinosissima |
| *R. micranthosepium* | unknown | Bred by Unknown (before 1800) |
| *R. Sanguinea* ‘Hybrid Gallica’ | unknown | Bred by Unknown (before 1820) |


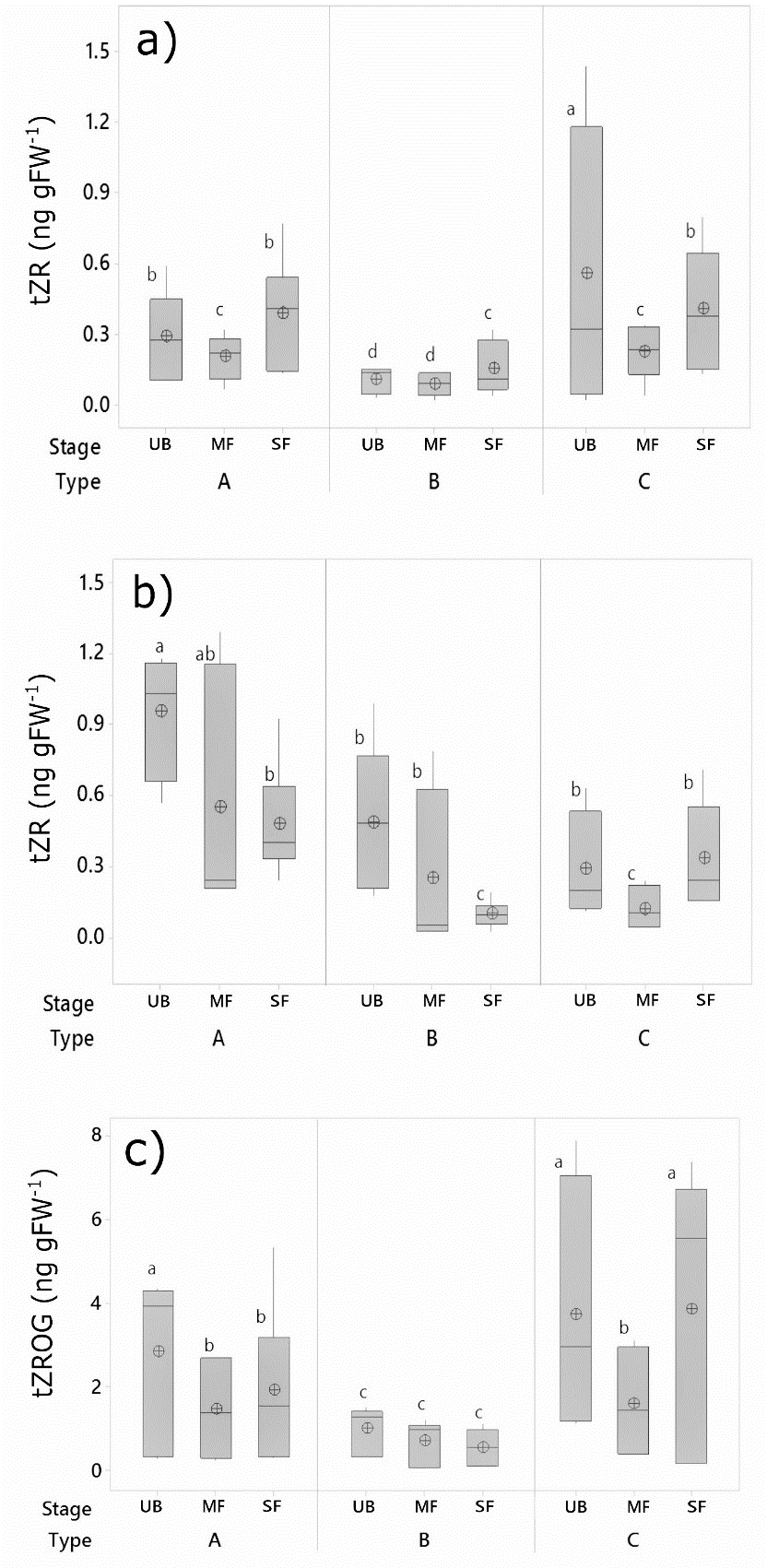


**Figure S1: Selected individual cytokinins in wild rose petals** (at stage UB= unopened buds, stage MF =open mature flower, stage SF=senescent flower) (a) tz = trans-zeatin, (b) tZR = trans-zeatin riboside, (c) tZROG = trans-zeatin riboside-o-glucoside. Bar graphs represent data as in Fig. S1. (3 species per monoterpene profile type were used, see methods). Equality of means is tested using GLM ANOVA followed by GH test (α=0.05) and significant differences are marked with unique letters.
